# Main considerable factors for correct laboratory test interpretation under DOA treatment

**DOI:** 10.1186/1477-9560-11-22

**Published:** 2013-11-01

**Authors:** Helen Mani, Edelgard Lindhoff-Last

**Affiliations:** 1 Department of Internal Medicine, Division of Vascular Medicine and Hemostaseology, Johann Wolfgang Goethe-University Hospital, Frankfurt/Main, Germany

## Abstract

**Summary:**

To avoid misinterpretation and mismanagement clinicians should be aware of the interference of new direct oral anticoagulants (DOA) on coagulation assays. A variety of oral anticoagulants targeting specific coagulation factors has already entered the market, and new indications for DOA will be released each year over the next few years. Due to their heterogeneous mode of action and different pharmacokinetic profile each DOA will vary in its effects on coagulations assays, and it is of current importance to recognize these variable effects.

In this summary the main considerable factors for correct laboratory test interpretation under DOA treatment are described.

## Introduction

In multiple clinical situations, coagulation parameters as the prothrombin time (PT) are checked for safe and effective treatment with vitamin K anticoagulants [1]. Global coagulation assays are performed on a routine base even after administration of the new direct oral anticoagulants (DOA), although routine laboratory monitoring is not required [2,3] for these new drugs as dabigatran, rivaroxaban or apixaban. Prolongation of clotting times after DOA administration may raise concerns about the anticoagulant treatment with DOA. As a result, data might be misinterpreted; DOA therapy might be discontinued; and another anticoagulant might be started.

Therefore, the main considerable factors for correct laboratory test interpretation under DOA treatment have to be known.

### Reason for interference of DOA on coagulation assays

**Figure 1:**
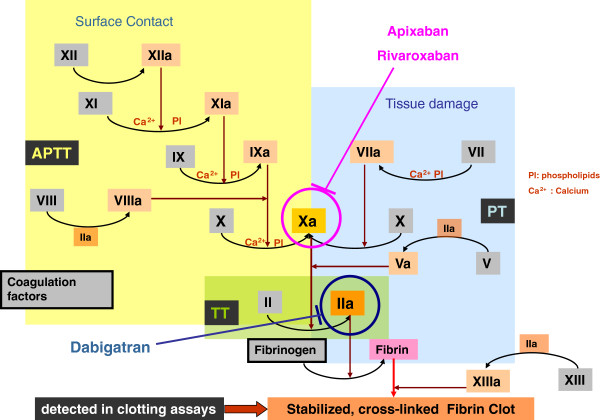
Classical coagulation pathway with clotting assays and targets of DOA.

Usually, the time between the addition of a coagulation activating reagents and the formation of the fibrin clot is measured. Increasing viscosity in the plasma sample allows the detection of the end point. Inhibition of Factor Xa results in inhibiting the formation of thrombin. Thrombin itself is needed to format the fibrin clot, which is detected in a coagulation clotting assay. Because rivaroxaban and apixaban are selective direct inhibitors of Factor Xa and dabiagtran a direct thrombin inhibitor, coagulation clotting assays are influenced by these drugs (Figure 1).

The in vitro and ex-vivo influence of rivaroxaban and dabigatran on global and specific coagulation assays are well described [4-13], since these two new oral anticoagulants are increasingly used for various indications worldwide. Moreover, apixaban, as second direct oral Factor Xa inhibitor, has recently received its approval for the prevention of stroke in patients with non-valvular atrial fibrillation, and its effect on routine and specific coagulation assays has been analysed, too [14]. As seen in Table 1, coagulation clotting assays are influenced by DOA to different extends.

**Table 1:** Influence of DOA on different coagulation assays

**Influence of DOA on coagulation parameters**			
PT [INR or sec]	↑	↑↑↑	↑↑
aPTT	↑↑↑	↑↑	↑
Thrombin Time	↑↑↑↑↑	-	-
Fibrinogen (Clauss method) derived Fibrinogen	↓(↓)	-	-
	(↑)	↑	(↑)
Antithrombin FXa-based assay	-	↑	↑
FIIa-based assay	↑	-	-
Intrinsic coagulation factors VIII, IX, XI, XII (based on aPTT)	↓↓	↓	↓
Extrinisic coagulation factors II,V,VII,X (based on PT)	↓	↓↓	↓↓
Factor XIII -photometric	↓↓	-	-
-immunologic	-	-	-
Protein S (clotting)	↑↑	↑↑	↑
Free Protein S antigen (immunologic)	-	-	-
(chromogenic)	↑↑	↑	↑
	-	-	-
Diluted Russel’s Viper Venom - test	↑	↑↑↑	↑↑
Activated Protein C-Ratio (based on aPTT-assay)	↑↑	↑	↑
Ecarin Clotting Time	↑↑	-	-

The results of the ex-vivo and in-vitro effects of these new drugs on the coagulation parameters as PT or aPTT can be used to find out the main considerable factors for correct interpretation of laboratory test results in patients under DOA treatment.

### The main considerable factors for correct laboratory test interpretation under DOA treatment

**Figure 2:**
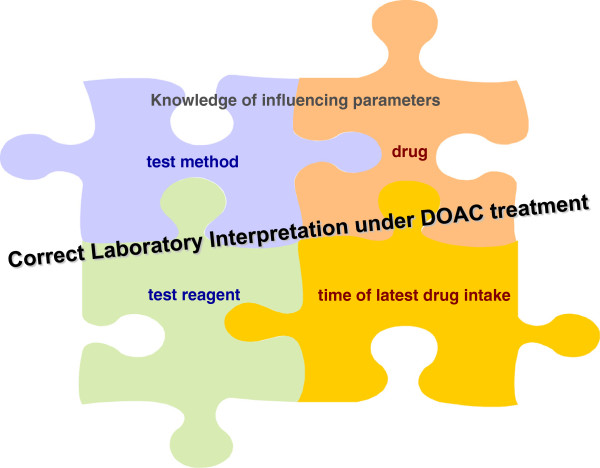
The four main considerable factors for correct laboratory test interpretation under DOA treatment.

To avoid erroneous interpretation it is important to be aware of the four main factors that have to be considered at the same time when coagulation parameters are measured in plasma with DOA (Figure 2).

1. Dependence on the drug.

Different mode of action – different effects on coagulation assays

**Figure 3:**
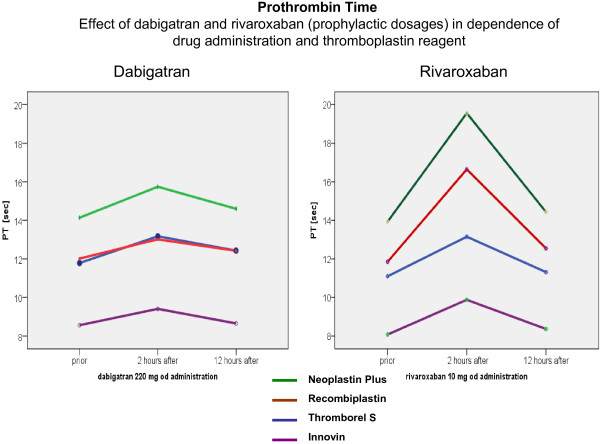
Effect of dabigatran and rivaroxaban on the prothrombin time in dependence of drug administration and thromboplastin reagent.

Rivaroxaban and apixaban as selective direct inhibitors of factor Xa, inhibit prothrombinase-bound factor Xa. Since the PT assay is sensitive to the prothrombinase complex, the PT is influenced by direct factor Xa inhibitors of factor Xa, whereas dabigatran in low doses has little effect on PT (Figure 3).

**Figure 4:**
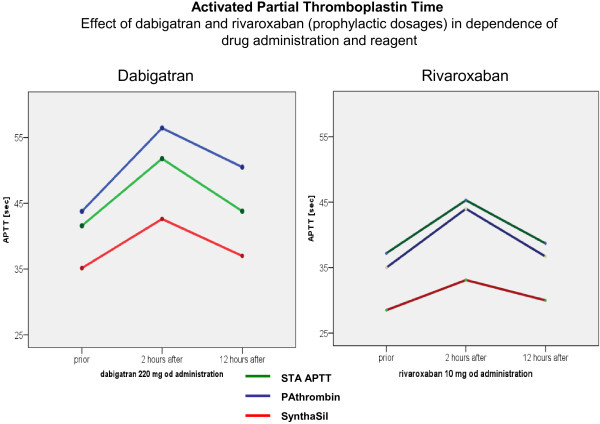
Effect of dabigatran and rivaroxaban on the activated partial thromboplastin time in dependence of drug administration and reagent.

As screening assay the aPTT detects abnormalities in kininogen, prekallikrein, XII, XI, IX, VIII, V, and factor Xa and thrombin, both targets of rivaroxaban and dabigatran. A prolongation of aPTT is observed in the presence of increasing doses of dabigatran and –to a much less extend – with rivaroxaban or apixaban [4,6,8,9,14,15] (Figure 4).

The thrombin time (TT) is used to measure fibrin polymerisation and is performed by adding a low concentration of thrombin to plasma. Since the TT assay is a direct measurement of thrombin activity, and dabigatran is a direct thrombin inhibitor, the TT assay is highly sensitive to dabigatran Table 1. TT is reported to response even at lowest dabigatran plasma concentrations [8,9,11,15]. Because rivaroxaban and apixaban do not directly inhibit thrombin, no influence of both drugs on TT is reported [7,11,14].

Clinicians should find out which drug was administrated to the patient before routine coagulation parameters are checked and interpreted.

2. Dependence on the drug pharmacokinetics and drug concentration.

More drug concentration in plasma-more effect on coagulation assays

Dabigatran, rivaroxaban and apixaban have predictable pharmacokinetic profiles with plasma concentrations reaching a peak within 2-4 hours after intakeand terminal half-lives of 7-17 hours. Corresponding to the half-lives, a decline of plasma concentration is observed over time; and consequently the effects of DOA on coagulation parameters also decrease over time. Since there is this direct correlation between drug concentration and its effect on clotting assays, it is of prime importance to know the time of drug intake in relation to blood sampling as can be seen for example in the values of PT and aPTT in Figures 3 and 4.

The prolongations of PT and aPTT are short lived. Two to three hours after DOA administration, a significant prolongation expressed in seconds of PT or aPTT will be observed. The minimum effect of DOA is observed directly before drug intake.

Therefore it is recommended, that laboratory monitoring of coagulation factors, if needed, should be performed directly before drug administration to keep the interaction of drugs low.

3. Dependence on the test method used in the laboratory.

Different test methods – different test results

The knowledge of the test method used for determination of the coagulation parameter is important for correct interpretation of results. Two examples:

a. Antithrombin determination can be performed using test systems, in which the plasma sample is incubated either with an excess of thrombin, or with an excess of factor Xa. Antithrombin levels based on addition of factor Xa are measured falsely high when rivaroxaban plasma samples are investigated. No influence is observed by rivaroxaban, when determination of antithrombin levels is based on thrombin [7].

b. The frequently used method for the determination of fibrinogen as one of the routine parameters in coagulation testing is the method according to Clauss. This method is based on the addition of an excess of thrombin to plasma. Rivaroxaban, does not influence fibrinogen assays based on the Clauss method because it does not inhibit thrombin activity [7]. Another method for measurement of fibrinogen is the PT derived fibrinogen assay as widely used alternative method, due to the fact that the total increase of turbidity during PT is directly proportional to the concentration of fibrinogen. The effect of rivaroxaban on PT derived fibrinogen assays varies significantly depending on the drug concentration and the PT reagent used for this assay [7].

Therefore, clinicians should to be aware of various effects of DOA depending on the test method. Dabigatran as direct thrombin inhibitor, influences fibrinogen assays based on Clauss method at increasing drug concentrations and test reagents- and to much less extend PT derived fibrinogen assays [16].

4. Dependence on the test reagent used in the laboratory.

Different test reagents – different influence of DOA

Several ex-vivo and in-vitro studies have shown that the prolongation of PT and aPTT depends on the different sensitivities of the thromboplastin reagents and APTT reagents used for the assay [6-12]. For example: different thromboplastin reagents exhibit different sensitivities to rivaroxaban at the same concentrations. A sensitive reagent such as Neoplastin Plus® has been significantly influenced by rivaroxaban while hardly any effect is observed on Innovin® (see Figure 2).

Therefore, clinicians should also be aware of the different sensitivities of DOA on various test reagents within one test parameter.

## Conclusion

In emergency situations like acute bleeding the clinicians could be misled by the results of coagulation tests, which are affected depending on various factors as the pharmacokinetics of the drug after intake or the type of test assay used.

For correct interpretation of coagulation assays during DOA treatment, clinicians need to know about the temporal effects of the target specific drugs on coagulation tests and the pitfalls in the laboratory.

The results of coagulation assays may differ from laboratory to laboratory depending on the test reagent and test method used in the laboratory, making comparisons between the laboratories difficult.

In future, practical approach in the laboratory is necessary and a comprehensive system of optimal patient care by exact interpretation of coagulation results under DOA treatment has to be developed worldwide in future.

## Competing interests

Prof Lindhoff-Last received honoraria for lecturing and consulting fees within the last 10 years from Bayer HealthCare, Boehringer Ingelheim, Pfizer-BMS, Roche Diagnostics, Siemens Diagnostics, Instrumentation Laboratory.

Dr. Mani declares any conflict of interest.

## Authors’ contributions

HM drafted the manuscript. ELL revised the manuscript. Both authors read and approved the final manuscript.
